# Supplementary material for: Children are not like other loads: a cross-cultural perspective on the influence of burdens and companionship on human walking
Source: PeerJ. 2018 Sep 12;6:e5547. doi: 10.7717/peerj.5547 (PMC6139008; doi:10.7717/peerj.5547)
Supplement: Table S1 [file peerj-06-5547-s001.docx]

Supplemental Table 1: Results of a Univariate GLM testing speed differences between the two locations.

| **Between-Subjects Factors** | | | |
| --- | --- | --- | --- |
|  | | Value Label | N |
| Location | 0 | Central Uganda | 969 |
|  | 1 | W.C. United States | 752 |
| Dyad Sex | 0 | Male | 692 |
|  | 1 | Female | 1029 |

| **Tests of Between-Subjects Effects** | | | | | |
| --- | --- | --- | --- | --- | --- |
| Dependent Variable: Speed (m/sec) | | | | | |
| Source | Type III Sum of Squares | df | Mean Square | F | Sig. |
| Corrected Model | 5.717 | 3 | 1.906 | 61.018 | .000 |
| Intercept | 1316.631 | 1 | 1316.631 | 42157.649 | .000 |
| Location | .860 | 1 | .860 | 27.526 | .000 |
| DyadSex | 1.379 | 1 | 1.379 | 44.152 | .000 |
| Location * DyadSex | 3.627 | 1 | 3.627 | 116.143 | .000 |
| Error | 53.624 | 1717 | .031 |  |  |
| Total | 1426.493 | 1721 |  |  |  |
| Corrected Total | 59.341 | 1720 |  |  |  |
|  | | | | | |
